# Supplementary material for: The utility of borescope and ATP Biofluorescence for inspection of ophthalmic phaco handpiece lumen: a single-center observational study
Source: PLoS One. 2025 May 8;20(5):e0323327. doi: 10.1371/journal.pone.0323327 (PMC12061102; doi:10.1371/journal.pone.0323327)
Supplement: Attachment — Submitted filename: PONE-D-24-50911.docx [file pone.0323327.s001.docx]

PLOS ONE

The utility of borescope and ATP Biofluorescence for inspection of ophthalmic phaco handpiece lumen: a single-center observational study

--Manuscript Draft--

| Manuscript Number: | PONE-D-24-50911 |
| --- | --- |
| Article Type: | Research Article |
| Full Title: | The utility of borescope and ATP Biofluorescence for inspection of ophthalmic phaco handpiece lumen: a single-center observational study |
| Short Title: | The utility of borescope and ATP Biofluorescence for inspection of ophthalmic phaco handpiece lumen |
| Corresponding Author: | Zhuoya YAO  Henan Provincial People's Hospital Zhengzhou, CHINA |
| Keywords: | Hand pieces; Visual inspection; Corrosion; Toxic anterior segment syndrome; patient safety |
| Abstract: | The presence of residual debris in the phaco handpiece lumen is implicated in the onset of intraocular inflammation. While ATP bioluminescence testing is accepted for evaluating cleanliness of surgical instruments, the borescope's ability to facilitate a visual inspection of the internal integrity of phaco handpiece lumens remains underreported. This study aims to evaluate the efficacy of current cleaning protocols and explore the borescope's utility in visually inspecting phaco handpieces. In this analysis, 41 phaco handpieces underwent borescope examination and ATP bioluminescence testing following thorough cleaning, with two inspections completed on each handpiece. Borescope inspections revealed that 56.10% of phaco handpieces harbored foreign materials or exhibited various forms of structural damage such as corrosion, rust, green lint, and discoloration. However, ATP bioluminescence testing deemed the cleanliness of all handpieces to be satisfactory. This study demonstrates the value of a borescope in the visual inspection of phaco handpieces, revealing issues such as corrosion and rust which necessitated immediate flushing of the lumen with sterile distilled or deionized water. The detection of green lint led to the prohibition of placing textile fibre-containing sterile towels on the operating table surface by sterile supply staff, enhancing patient safety measures. Future efforts will focus on addressing the challenges posed by corrosion and particulate formation during rinsing. |
| Order of Authors: | Meng Zhan |
|  | Zhuoya YAO |
|  | Junhui Geng |
|  | Manchun Li |
|  | Lina Ding |
| Additional Information: |  |
| Question | Response |
| Financial Disclosure  Enter a financial disclosure statement that describes the sources of funding for the work included in this submission. Review the [submission guidelines](http://journals.plos.org/plosone/s/submission-guidelines#loc-financial-disclosure-statement) for detailed requirements. View published research articles from [*PLOS ONE*](https://journals.plos.org/plosone/) for specific examples. | The author(s) received no specific funding for this work. |

| This statement is required for submission and will appear in the published article if the submission is accepted. Please make sure it is accurate. |  |
| --- | --- |
| Funded studies  Enter a statement with the following details:   - Initials of the authors who received each award - Grant numbers awarded to each author - The full name of each funder - URL of each funder website - Did the sponsors or funders play any role in the study design, data collection and analysis, decision to publish, or preparation of the manuscript? |  |
| Did you receive funding for this work? |  |
| Competing Interests  Use the instructions below to enter a competing interest statement for this submission. On behalf of all authors, disclose any [competing interests](http://journals.plos.org/plosone/s/competing-interests) that could be perceived to bias this work—acknowledging all financial support and any other relevant financial or non- financial competing interests.  This statement is required for submission and will appear in the published article if the submission is accepted. Please make sure it is accurate and that any funding sources listed in your Funding Information later in the submission form are also declared in your Financial Disclosure statement.  View published research articles from  [*PLOS ONE*](http://journals.plos.org/plosone/) for specific examples. | The authors have declared that no competing interests exist. |

| NO authors have competing interests  Enter: *The authors have declared that no competing interests exist.*  Authors with competing interests  Enter competing interest details beginning with this statement:  *I have read the journal's policy and the authors of this manuscript have the following competing interests: [insert competing interests here]* |  |
| --- | --- |
| * typeset |  |
| Ethics Statement  Enter an ethics statement for this submission. This statement is required if the study involved:   - Human participants - Human specimens or tissue - Vertebrate animals or cephalopods - Vertebrate embryos or tissues - Field research   Write "N/A" if the submission does not require an ethics statement.  General guidance is provided below. Consult the [submission guidelines](http://journals.plos.org/plosone/s/submission-guidelines#loc-guidelines-for-specific-study-types) for detailed instructions. Make sure that all information entered here is included in the Methods section of the manuscript. | N/A |

| Format for specific study types  Human Subject Research (involving human participants and/or tissue)   - Give the name of the institutional review board or ethics committee that approved the study - Include the approval number and/or a statement indicating approval of this research - Indicate the form of consent obtained (written/oral) or the reason that consent was not obtained (e.g. the data were analyzed anonymously)   Animal Research (involving vertebrate animals, embryos or tissues)   - Provide the name of the Institutional Animal Care and Use Committee (IACUC) or other relevant ethics board that reviewed the study protocol, and indicate whether they approved this research or granted a formal waiver of ethical approval - Include an approval number if one was obtained - If the study involved *non-human primates*, add *additional details* about animal welfare and steps taken to ameliorate suffering - If anesthesia, euthanasia, or any kind of animal sacrifice is part of the study, include briefly which substances and/or methods were applied   Field Research  Include the following details if this study involves the collection of plant, animal, or other materials from a natural setting:   - Field permit number - Name of the institution or relevant body that granted permission |  |
| --- | --- |
|  |  |
| Data Availability  Authors are required to make all data underlying the findings described fully available, without restriction, and from the time of publication. PLOS allows rare exceptions to address legal and ethical concerns. See the [PLOS Data Policy](http://journals.plos.org/plosone/s/data-availability) and [FAQ](http://journals.plos.org/plosone/s/data-availability#loc-faqs-for-data-policy) for detailed information. | Yes - all data are fully available without restriction |

| A Data Availability Statement describing where the data can be found is required at submission. Your answers to this question constitute the Data Availability Statement and will be published in the article, if accepted. |  |
| --- | --- |
| Important: Stating ‘data available on request from the author’ is not sufficient. If your data are only available upon request, select ‘No’ for the first question and explain your exceptional situation in the text box. |  |
| Do the authors confirm that all data underlying the findings described in their manuscript are fully available without restriction? |  |
| Describe where the data may be found in full sentences. If you are copying our sample text, replace any instances of XXX with the appropriate details.   - If the data are held or will be held in a public repository, include URLs, accession numbers or DOIs. If this information will only be available after acceptance, indicate this by ticking the box below. For example: *All XXX files are available from the XXX database (accession number(s) XXX, XXX.*). - If the data are all contained within the manuscript and/or Supporting Information files, enter the following: *All relevant data are within the manuscript and its Supporting Information files.* - If neither of these applies but you are able to provide details of access elsewhere, with or without limitations, please do so. For example:   *Data cannot be shared publicly because of [XXX]. Data are available from the XXX Institutional Data Access / Ethics Committee (contact via XXX) for researchers who meet the criteria for access to confidential data.*  *The data underlying the results presented in the study are available from (include the name of the third party* | All relevant data are within the manuscript and its Supporting Information files. |

| *and contact information or URL).*   - This text is appropriate if the data are owned by a third party and authors do not have permission to share the data.   * typeset |  |
| --- | --- |
| Additional data availability information: | Tick here if the URLs/accession numbers/DOIs will be available only after acceptance of the manuscript for publication so that we can ensure their inclusion before publication. |

[Click here to access/download;Manuscript;Manuscript.docx](https://www2.cloud.editorialmanager.com/pone/download.aspx?id=38194809&guid=6b79e060-a19d-454f-b85d-80a63129668d&scheme=1)[<https://www2.cloud.editorialmanager.com/pone/download.aspx?id=38194809&guid=6b79e060-a19d-454f-b85d-80a63129668d&scheme=1>](https://www2.cloud.editorialmanager.com/pone/download.aspx?id=38194809&guid=6b79e060-a19d-454f-b85d-80a63129668d&scheme=1)

### 1 The utility of borescope and ATP Biofluorescence for inspection of ophthalmic

2 **phaco handpiece lumen: a single-center observational study**

3

4	Zhan meng^1◻^, Yao zhuoya^1,2*◻^,,Geng junhui^1^＆, Li manhucn^1^＆, Ding lina^1^

5

6 ^1^Central Sterile Supply Department,Henan Provincial People's Hospital, Zhengzhou
7 University People's Hospital, Zhengzhou, China
8 ^2^Henan Provincial Medical Key Laboratory for Quality Control of Medical Devices
9 Sterilization, Zhengzhou, China

10

11 *Corresponding author
12 E-mail: [zm15093137681@sina.com](mailto:zhuoya%2Czm15093137681@sina.com)

13

14 ◻These authors contributed equally to this work.
15 ＆These authors also contributed equally to this work.

16

17

18

19

20

21

22

23

24

25

26

27

28

29

30

31	**Abstract**

32 The presence of residual debris in the phaco handpiece lumen is implicated in the
33 onset of intraocular inflammation. While ATP bioluminescence testing is accepted for
34 evaluating cleanliness of surgical instruments, the borescope's ability to facilitate a
35 visual inspection of the internal integrity of phaco handpiece lumens remains
36 underreported. This study aims to evaluate the efficacy of current cleaning protocols
37 and explore the borescope's utility in visually inspecting phaco handpieces. In this
38 analysis,	41	phaco	handpieces	underwent	borescope	examination	and ATP
39 bioluminescence testing following thorough cleaning, with two inspections completed
40 on each handpiece. Borescope inspections revealed that 56.10% of phaco handpieces
41 harbored foreign materials or exhibited various forms of structural damage such as
42 corrosion, rust, green lint, and discoloration. However, ATP bioluminescence testing
43 deemed the cleanliness of all handpieces to be satisfactory. This study demonstrates the
44 value of a borescope in the visual inspection of phaco handpieces, revealing issues such
45 as corrosion and rust which necessitated immediate flushing of the lumen with sterile
46 distilled or deionized water. The detection of green lint led to the prohibition of placing
47 textile fibre-containing sterile towels on the operating table surface by sterile supply
48 staff, enhancing patient safety measures. Future efforts will focus on addressing the
49 challenges posed by corrosion and particulate formation during rinsing.

50

51

52

53

54

55

56

57

58

59

# 60 Introduction

61 The prevalence of ophthalmic surgeries has notably increased following advancements
62 in diagnostic and therapeutic ophthalmology. Cataract surgery, a major ocular
63 procedure, utilizes the smallest intraocular surgical instruments. While these
64 instruments are typically minimally contaminated by tissues or bacteria [1], even trace
65 amounts of residual detergents or chemical pollutants, otherwise tolerable in other body
66 sites, can provoke severe consequences within the eye, leading to the toxic anterior
67 segment syndrome (TASS) [2,3].
68 TASS, a non-infectious postoperative inflammatory condition, is commonly
69 observed following cataract surgery, but can also develop following corneal
70 transplantation and posterior segment operations [4-7]. Recent studies have identified
71 inadequate flushing, enzymatic detergent usage, metals, viscoelastic contamination,
72 and adverse drug reactions as the primary contributors to TASS development [8-10].
73 Comprehensive clinical and laboratory examinations often fail to identify pathogens in
74 TASS case [1,11], highlighting the critical role of thorough instrument cleaning and
75 sterilization practices in preventing TASS.
76 The ophthalmic phaco handpiece, a key tool in cataract surgery, emulsifies the lens
77 nucleus into a form that can be aspirated. If the residual lens cortex and viscoelastic
78 materials are inadequately cleaned from the phaco handpiece, they may transform into
79 toxic agents during sterilization, leading to intraocular toxicity [12]. Guidelines for
80 cleaning and sterilization of intraocular surgical instruments state that instruments must
81 be visually inspected for debris and damage after cleaning and before packaging for
82 sterilization to ensure that debris removal is complete [1]. While the utility of
83 borescopes in inspecting endoscopes is well-documented, highlighting their importance
84 for identifying damage, abnormalities, and residual materials [13,14], evidence of their
85 use in phaco handpiece inspections remains sparse.
86 To enhance the cleaning efficacy of phaco handpieces, our Central Sterile Supply
87 Department (CSSD) utilized both a borescope and ATP bioluminescence for lumen
88 assessments. The study primarily aimed to: 1) evaluate the borescope's effectiveness in
89 facilitating the visual inspection of phaco handpieces; and 2) scrutinize the CSSD’s
90 existing reprocessing protocols to determine opportunities for ongoing process
91 improvement.

92

93	**Methods**

## 94 Settings

95 The study was conducted in a large tertiary general hospital and its associated eye
96 hospital, which houses 12 ocular surgery suites with an annual surgical volume of over
97 20,000 cases, 184 open beds, and an annual outpatient volume of nearly 500,000. In
98 this setting, all used ophthalmic surgical instruments, including phaco handpieces, are
99 centrally processed in the CSSD. The study did not involve human research subjects.

## 100 Phaco handpieces

101 We utilize titanium alloy phaco handpieces (model BL3170, Bausch & Lomb) in our
102 institution. It has two lumens: one for irrigation and the other for aspiration. The
103 cleaning of phaco handpieces is conducted manually. Initially, these instruments are
104 pretreated by nurses in the ophthalmic operating room before being transported to the
105 CSSD in a sealed transfer box. Within the designated ophthalmic processing area of the
106 CSSD, the staff undertakes the cleaning process adhering to the manufacturer’s
107 recommendations. This process involves rinsing the surface of the instrument with
108 running water, followed by the insertion and retraction of a 50 ml syringe into both the
109 irrigation and aspiration lumens of the phaco handpiece to ensure thorough internal
110 cleaning. Subsequently, the handpieces are immersed in an enzyme-free detergent
111 solution, with particular attention given to scrubbing the lumens and threads with a
112 brush beneath the liquid surface to remove any adherent residues. Following this
113 cleaning, a comprehensive rinse is performed, culminating in a final rinse to ensure the
114 elimination of all residual substances. The subsequent phase involves both lumens
115 being subjected to air-drying by utilizing jet guns, each for a duration of 30 seconds, to
116 remove any residual moisture. The cleanliness of the handpieces is then evaluated
117 through both borescope inspections and ATP bioluminescence testing to ascertain the
118 efficacy of the cleaning process.

## 119 Borescope Inspection

120 The apparatus (Shenzhen Xinruida) comprises a tablet PC running proprietary software
121 and a borescope with an operating handle. A data cable is used to connect the two
122 components. A 1.2-meter-long, 1.8-millimeter-diameter, high-precision borescope with
123 a lens resolution of up to 300,000 pixels is inserted into the instrument's lumen by the
124 examiner, who also uses the handle to take pictures and videos and the tablet to visualize
125 them. In this project, borescope inspections are systematically carried out by the CSSD
126 staff, with the examination frequency based on the daily procedural workload, ensuring
127 a broad temporal spread for accuracy in results. The examiner first inserts the borescope
128 into the inlet of the irrigation lumen until reaching the end of the lumen. Thereafter,
129 insertion into the aspiration lumen is performed. Because of the inner diameter of the
130 aspiration lumen, only the inlet and outlet regions are inspected. Each handpiece
131 inspection lasted approximately 3 minutes, with the borescope sanitized using 75%
132 ethanol between uses. Notably damaged handpieces were identified for subsequent
133 repair.

## 134 ATP Biofluorescence

135 The Association	for	the Advancement	of	Medical	Instrumentation	(AAMI)
136 recommends that instruments must be verified by a cleaning compliance test after
137 finishing the cleaning procedure [15]. ATP Biofluorescence is a fast and reliable test for
138 quantitative assessment of cleaning quality. We followed the standard operating
139 procedure utilizing a liquid sampling swab from 3M™ Clean-Trace™. During the
140 process, the phaco handpiece was held vertically to facilitate the filling of both the
141 irrigation and aspiration tube lumens with approximately 10 ml of sterile water each by
142 CSSD staff. This sterile water was then collected into a sterile container under the phaco
143 handpiece. Care was taken to moisten the swab head with the rinse solution collected
144 in the sterile container without touching the inner walls of the sterile container. Once
145 moistened, the swab was transferred to a test tube, agitated, and then introduced into
146 the ATP biofluorescence detector for analysis. According to the testing protocol, a
147 reading of ≤150 Relative Light Units (RLU) indicated satisfactory cleaning.

148

149	**Results**

## 150 Phaco handpieces characteristics and overall findings

151 In total, 41 phaco handpieces were subjected to both examinations, with a service life
152 of 2-8 years and a mean value of 5.39±1.61 years. Eighteen cases passed all inspections,
153 17 cases had one finding in the borescopes's visual inspection, and six handles had
154 multiple abnormal findings (Table 1). The difference in the number of years of usage
155 between the handles was statistically significant (*F*=7.384, *p*<0.05).

156

### 157 Table 1: A comparison of the number of significant findings for phaco handpieces*

| **Groups** | **Cases** | **Age (years)** | ***F*** | ***P-*value** |
| --- | --- | --- | --- | --- |
| No finding | 18 | 4.44±1.69 |  |  |
| One finding | 17 | 6.06±1.03 | 7.384 | 0.002 |
| Multiple findings | 6 | 6.33±1.37 |  |  |
| Total | 41 | 5.39±1.61 |  |  |

158 *As all phaco handpieces passed the ATP Biofluorescence assay, the numbers in the
159 table are the result of visual inspection by borescopes.

160

## 161 Borescopes Inspection

162 In 23 of the cases (56.10%), the borescope inspections of phaco handpieces revealed
163 significant findings within the lumens, such as corrosion, rust, green lint, and
164 discoloration (Table 2). Up to 53.66% of the irrigation lumens of the phaco handpieces
165 were problematic, suggesting a significant difference compared to the aspiration lumens
166 (*χ^2^*=12.004, *p* < 0.05).

### 167 Table 2 Comparing the number of results in each one of the various lumens of

168 **the phaco handpieces***

Site	Finding Items*(%)	Total	*χ^2^	P* value

|  | Corrosion | Rust | Green lint | Discolouration |  | | |
| --- | --- | --- | --- | --- | --- | --- | --- |
| Irrigation lumen ◻n=41◻ | 10(24.39%) | 7(17.07%) | - | 5(12.20%) | 22(53.66%) |  |  |
| Aspiration lumen◻n=41◻ | - | 5(12.20%) | 2 (4.88%) | - | 7 (17.08%) | 12.004 | 0.001 |

169 *These results represent objective findings only and are not indicative of severity.

170

## 171 Findings within the irrigation lumens

172 Pitting corrosion of varying degrees was observed in 10 cases (24.39%) within the
173 irrigation lumen, predominantly at the interface, mid-lumen, and outlet, appearing as
174 flaky formations (Fig 1 A1-2). Rust corrosion was detected in 7 cases (17.07%), mainly
175 at the articulation threads (Fig 1 B1-2). Discoloration along the lumen’s interior wall
176 was noted in 5 cases (12.20%) (Fig 1 D1-2).

## 177 Findings within the aspiration lumens

178 Rust was identified at the interface and articulation of the aspiration lumen in 5 cases
179 (12.20%). Notably, green lint was discovered within the aspiration lumen in 2 instances
180 (4.88%) (Fig 1 C1-2), likely originating from sterile wipes made of textile material used
181 on the operating table, which could have entered the lumen during air drying. This
182 finding led to the cessation of textile sterile wipe use on the operating table to prevent
183 potential ocular washout risks.

184

### 185 Fig 1 Examples of borescope findings in the phaco handpieces

186 (A1-3: pitting in the form of flakes; B1-3: rust; C1-2: green lint at the entrance to the
187 aspiration lumen; D1-2: discoloration)

188

## 189 ATP Biofluorescence

190 The ATP biofluorescence tests for all 41 phaco handpieces showed RLU values below
191 150, indicating satisfactory cleaning.

192

193	**Discussion**

194 The phaco handpiece, a critical tool in ophthalmic surgeries such as cataract removal,
195 demands rigorous cleaning procedures due to its complex internal structure. Our
196 Central Sterile Supply Department (CSSD) emphasizes the careful cleaning of these
197 handpieces, following specific protocols that include isolated cleaning processes,
198 designated processing areas, and the avoidance of enzyme-based detergents to
199 minimize risks arising from cross-contamination and residue.
200 Traditionally, handpiece inspections have relied on external examinations utilizing
201 light-ray magnifying glasses, which are less effective for assessing the narrow internal
202 lumen. As a result, quantitative methods such as ATP bioluminescence and residual
203 protein testing have become standard for evaluating lumen cleanliness [15]. In this
204 study, we incorporated ATP bioluminescence testing alongside borescope examinations,
205 providing a detailed view of the internal condition of the handpieces, thus bridging the
206 gap between superficial and thorough internal assessments.
207 The study identified pitting corrosion in 24.39% of the handpieces, potentially
208 related to the balanced salt solutions used for irrigation during surgery; these contain
209 electrolytes that could exacerbate corrosion over time [16]. Furthermore, 12.20% of the
210 handpieces exhibited rust, particularly at the entry and articulation points, raising
211 concerns about bacterial adhesion and biofilm formation on the instrument surfaces.
212 Instrument corrosion and rust production is a slow process, and "particle" formation
213 can be seen in the initial stages of this process [17]. Many scholars who have found
214 metallic fragments in the eye have reported that the main component of the metal
215 fragments was titanium, which is consistent with the composition of the handpieces and
216 tips [18,19,21]. Martinez-Toldos et al. [19] found that the vibration of the phaco
217 handpiece, which is subjected to frequent use, can lead to the dislodgement of these
218 metal particles into the eye due to the wear and tear of the handles. In previous cases,
219 metal fragments were well tolerated in the eye, but a case report described recalcitrant
220 postoperative inflammation due to metal fragments on the surface of the iris [20]. Paolo
221 et al. [21] suggested that even if sterile, the fragments can potentially trigger
222 inflammation and lead to TASS. However, there is controversy regarding the origin and
223 hazardous effects of these metal particles. In response to these findings, we advised
224 ophthalmic operating theatre nurses to promptly rinse the handpieces with sterile
225 distilled or deionized water post-surgery to minimize electrolyte residue. Additionally,
226 the discovery of green lint in the aspiration lumen prompted a change in drying
227 practices, specifically prohibiting the use of textile sterile wipes on the operating table
228 to avoid lint contamination in the handpieces.
229 Furthermore, the topic of instrument aging is broad. Poor functioning is associated
230 with instrument aging. However, corrosion, rust, and discoloration did not impair the
231 applicability of the instruments in this study. Malfunctioning ultrasound transducers,
232 connecting wires, and connectors are the primary causes of instrument repair[22]. After
233 5 years of use, we advised administrators should pay attention to phaco handpieces,
234 especially to their irrigation lumens, to promptly detect structural damages and to
235 ensure their safe usage.
236 While ATP bioluminescence testing yielded satisfactory results, confirming the
237 effectiveness of our cleaning procedures, the structural damage revealed by the
238 borescope underscored the ongoing risk of biofilm protection [17]. Thus, we advocate
239 for a combined approach to enhance the accuracy of our cleaning quality assessments,
240 utilizing both ATP bioluminescence for quantitative assessment and borescope
241 inspections for qualitative evaluation.

## 242 Strengths and limitations

243 Our study has some limitations. Firstly, the clinical significance of these structural
244 abnormalities remains unclear. Our hospital's assessments after cataract surgery did not
245 show any cases of intraocular metallic fragments. Nevertheless, these results should
246 serve as a helpful reminder of potential dangers. Secondly, this was a single-center study,
247 which should be expanded in the future to incorporate data from a larger sample size.

248

249	**Conclusion**

250 This study confirms the vital role of the borescope in examining phaco handpieces but
251 also facilitates enhancements in our cleaning protocols, significantly advancing patient
252 and handler safety. Future efforts will focus on addressing the challenges posed by
253 corrosion and particulate formation during rinsing, further refining our sterilization
254 practices.

255

# 256 Acknowledgements

257 We wish to thank the nurses in the Ophthalmic Operating Room of Henan Provincial
258 Eye Hospital for supporting this study. The authors also thank the CSSD staff of Henan
259 Provincial People's Hospital for their cooperation and support.

260

# 261 Supporting information

262 S1 Fig.1
263 (TIFF)
264 S2 Dataset.
265 (XLSX)

266

# 267 Author Contributions

268 **Conceptualization:**Zhan meng,Yao zhuoya,Geng junhui.
269 **Formal analysis:**Zhan meng,Yao zhuoya,Ding lina.
270 **Data curation:**Zhan meng,Geng junhui.
271 **Funding acquisition:**Geng junhui,Ding lina.
272 **Investigation:**Ding lina.
273 **Methodology:**Yao zhuoya.
274 **Supervision:**Li manchun,Ding lina.
275 **Validation:**Li manchun,Yao zhuoya.
276 **Writing original draft:**Zhan meng,Yao zhuoya.
277 **Writing-reviewing	and	editing:**Zhan	meng,Yao	zhuoya,Li	manchun,Geng
278 junhui,Ding lina.

### 279 References

280 1. Mamalis, N. and Chang, D.F. Guidelines for the cleaning and sterilization of
281 intraocular	surgical	instruments.J	Cataract	Refract	Surg.2018;44(6):	675-6.
282 PMID:30041738
283 2. Mamalis N. Toxic anterior segment syndrome: role of enzymatic detergents used in
284 the cleaning of intraocular surgical instruments. J Cataract Refract Surg.2016; 42:1249-
285 50. PMID:27697240
286 3. Parikh C, Sippy BD, Martin DF, Edelhauser HF. Effects of enzymatic steriliz
287 ation detergents on the corneal endothelium. Arch Ophthalmol 2002;120:165-72.
288 PMID:11831918
289 4. Maier P, Birnbaum F, Bohringer D, Reinhard, T. Toxic anterior segment syn
290 drome following penetrating keratoplasty. Arch Ophthalmol. 2008;126:1677-81.P
291 MID:19064848
292 5.Sevimli N, Karadag R, Cakici O, Bayramlar, H,Okumus, S,Sari, U. Toxic anterior
293 segment syndrome following deep anterior lamellar keratoplasty. Arq Bras Oftalmol.
294 2016;79:330-2.PMID:27982215
295 6.Park CY, Lee JK, Chuck RS. Toxic anterior segment syndrome-an updated review.
296 BMC Ophthalmol 2018;18:276.PMID:30359246
297 7. Patel SB, Reddy NK, He YG. Toxic Posterior Segment Syndrome after Dropless
298 Cataract	Surgery	with	Compounded	Triamcinolone-Moxifloxacin.	Retina.
299 2020;40:446-55.PMID:30689622
300 8.Bodnar Z, Clouser S, Mamalis N. Toxic anterior segment syndrome: Update on the
301 most common causes. J Cataract Refract Surg. 2012; 38:1902-10.PMID:22999515
302 9. Mamalis N. Toxic anterior segment syndrome. J Cataract Refract Surg. 2006;
303 32(2):181-2.PMID:16565012
304 10.Cutler Peck CM, Brubaker J, Clouser S, Danford C, Edelhauser HE, Mamalis N.
305 Toxic anterior segment syndrome: common causes. J Cataract Refract Surg.
306 2010;36(7):1073-80.PMID:20610082
307 11. Moyle W, Yee RD, Burns JK, Biggins T. Two consecutive clusters of toxic anterior
308 segment syndrome. Optom Vis Sci. 2013;90(1):e11–23.PMID:23271338
309 12. Ma Y, He J. Research progress of ocular ganglion toxic reaction s
310 yndrome. Int. J. Ophthalmol.2017;17(4):669-72. DOI：10.3980/j.issn.1672-5123.
311 2017.4.20
312 13. Galdys AL, Marsh JW, Delgado E,Pasculle, AW,Pacey, M,Ayres, AM, et al.
313 Bronchoscope-associated clusters of multi-drugresistant Pseudomonas aeruginosa and
314 carbapenem-resistant Klebsiella pneumoniae. Infect Cont Hosp Ep. 2019;40:40-
315 46.PMID:30451128
316 14. Ofstead CL, Hopkins KM. Sterilization central: the value of borescopes in detecting
317 damage, soil, fluid, and foreign objects in flexible endoscopes. Biomed Instrum Technol.
318 2020;54:146-52.PMID:32186902
319 15. Association for the Advancement of Medical Instrumentation. ANSI/AAMI
320 ST79:2017. Comprehensive Guide to Steam Sterilization and Sterility Assurance
321 in Health Care Facilities. Arlington, VA: AAMI; 2017.https://[www.aami.org/sta](http://www.aami.org/sta)
322 ndards/featured-standards/ansi-aami-st79
323 16. Wallace, M.M,Keck, T,Dixon, H,Yassin, Ml.Borescope examination and microbial
324 culture results of endoscopes in a tertiary care hospital led to changes in storage
325 protocols to improve patient safety.Am J of Infect Control.2023;51(4): 361-6.
326 PMID:36122631
327 17. Jia FQ,Song M,Nie YL. Analysis of factors affecting corrosion of surgical
328 instruments	and	countermeasures.Nurs.	Res.2009;23(03):262-4.	DOI	：
329 10.3969/j.issn.1009-6493.2009.03.044
330 18. Braunstein RE, Cotliar AM, Wirostko BM,Gorman BD.Intraocular metallic-
331 appearing	foreign	bodies	after	phacoemulsification.J	Cataract	Refract	Surg.
332 1996;22:1247-50.PMID:8972380
333 19. Martinez-Toldos JJ, Elvira JC, Hueso JR, Artola A, Menguel E, Barcelo A, Garcia
334 J, Martinez-Reina MJ. Metallic fragment deposits during phacoemulsification. J
335 Cataract Refract Surg. 1998;24:1256-60.PMID:9768404
336 20. Stangos AN, Pournaras CJ, Petropoulos IK. Occult anterior-chamber metallic
337 fragment postphacoemulsification masquerading as chronic recalcitrant postoperative
338 inflammation. Am J Ophthalmol 2005; 139:541-2.PMID:15767070
339 21. Paolo C, Rossella DA, Manlio A, Gianluca T,Federica B, Elena M.Chemical and
340 physical analysis of phaco handpiece tip surfaces before and after cataract surgery.J
341 Cataract Refract Surg. 2017; 43:1107-14.PMID:28917414
342 22. Ling XX,Tang Y,Zhu MS,Zhang PH,LingXQ,Chen CF.Causes of Failure of Handle
343 of	Phacoemulsifier	and	Targeted	Measures.J	Nurs.2021;28(9):76-8.	DOI ：
344 10.16460/j.issn1008-9969.2021.09.076
345 23. Ding LN, Yao ZY, Geng JH,Zhan M,Li MC,Wang JF. Summary of the best evidence
346 for quality management of ophthalmic ultrasonic emulsion handle cleaning. Chin. Nurs.
347 Educ.2024;21(1):110-8.DOI：10.3761/j.issn.1672-9234.2024.01.018

348

349

350

351

352

353

354

355

356

357

358

359

360

361

362

363

364

365

366

367
