# Supplementary material for: The utility of borescope and ATP Biofluorescence for inspection of ophthalmic phaco handpiece lumen: a single-center observational study
Source: PLoS One. 2025 May 8;20(5):e0323327. doi: 10.1371/journal.pone.0323327 (PMC12061102; doi:10.1371/journal.pone.0323327)
Supplement: Attachment — Submitted filename: Response to Reviewers.docx [file pone.0323327.s003.docx]

**PONE-D-24-50911- "The utility of borescope and ATP Biofluorescence**

**for inspection of ophthalmic phaco handpiece lumen: a single-center**

**observational study"**

Dear Editors and Reviewers,

We appreciate the opportunity to revise our manuscript titled "The utility of borescope and ATP Biofluorescence for inspection of ophthalmic phaco handpiece lumen: a single-center observational study" and are grateful for the insightful comments provided by the reviewers. Those comments are all valuable and helpful for revising and improving our paper, as well as the important guiding significance to our research. In the following, we have provided detailed responses to each reviewer's comments. Revised portions are marked in red on the paper. Additionally, we have conducted a comprehensive revision of the entire manuscript. In this response letter, the reviewers' comments are presented in italics, and our corresponding changes and additions to the manuscript are highlighted in red text. We have tried our best to make all the revisions clear, and we hope that the revised manuscript meets the requirements for publication.

**Response to Reviewers**

***Reviewer #1: Mention guideline names for “instruments must be visually inspected for debris and damage after cleaning and before packaging for sterilization to ensure that debris removal is complete”.***

Response:

I apologize for any misinterpretation caused by my expression. This guideline is titled Guidelines for the Cleaning and Sterilization of Intraocular Surgical Instruments, and we specified it in the original article. It was created in collaboration with the Ophthalmic Instrument Cleaning and Sterilization (OICS) Task Force and published in 2018. This group, is made up of representatives of the American Society of Cataract and Refractive Surgery (ASCRS), the American Academy of Ophthalmology (AAO), and the Outpatient Ophthalmic Surgery Society (OOSS). These professional societies represent ophthalmologists and the clinical staff of ophthalmic outpatient surgery centers, including surgeons, nurses, and technicians. The OICS Task Force includes experts on TASS and endophthalmitis and has written this document to provide specialty-specific, evidence-based guidelines for the cleaning and sterilizing of intraocular surgical instruments. *(P3, Line 43-44)*

***Reviewer #2: Describe the functions of CSSD in the part of Introduction.***

Response:

Thanks for your suggestion. We have rewritten this part according to the Reviewer's suggestion. The Describe the functions of CSSD have been added to make the Introduction more detailed:” *The Central Sterile Supply Department (CSSD) in China oversees the maintenance of all reusable surgical instruments, including ophthalmic instruments. Its responsibilities encompass recovery, sorting, cleaning, disinfection, and sterilization of these instruments to ensure their sterility for reuse [12].” (P3, Line 35-39)*

*References*

*12.National Health and Family Planning Commission, People's Republic of China.WS310.1(R2016) Hospital central sterile supply department-Part 1: managementstandard.2017:1. Available from: http://www.nhc.gov.cn/fzs/s7852d/201701/b11cdd47e5624d698f0d1f3e25e0c9b8.shtml*

***Reviewer#3: Describe details about borescope and ATP bioluminescence in the part of Introduction.***

Response:

Thanks for your suggestion. We have rewritten this part according to the Reviewer's suggestion. The Describe the functions of CSSD have been added to make the Introduction more detailed:” *However, many modern surgical instruments feature complex designs with crevices, hinges, and narrow lumens that hinder the assessment of cleanliness through visual inspection alone [14]. The ophthalmic phaco handpiece presents a similar challenge due to its small lumen. Since the Adenosine Triphosphate (ATP) is found in all living tissues, its concentration serves as an indicator of bioburden, the greater the ATP level, the greater the contamination. ATP bioluminescence testing quantifies fluorescence intensity to estimate ATP levels [15], offering a sensitive and widely employed method for determining cleanliness in CSSDs. In recent years, borescopes have become increasingly utilized in the visual inspection of flexible endoscopes [16,17]. These small, light-equipped cameras allow CSSD staff to examine areas that are otherwise hidden from view, thereby providing an approach for directly assessing the internal cleanliness of lumened instruments [18]. (P3-4, Line 46-57)*

*References*

*14.Association for the Advancement of Medical Instrumentation. AAMI TIR30:2011(R2016).A Compendium Of Processes, Materials, Test Methods, And Acceptance Criteria For Cleaning Reusable Medical Devices. Arlington, VA: AAMI; 2011.Available from:https://array.aami.org/doi/book/10.2345/9781570204197*

*15.Fitts LN, Yegge J, Goris A, Vinson S, Dubberke E. How clean is	clean enough? An observational pilot study to assess central sterilization processing efficacy with adenosine triphosphate levels. Am J Infect	Control. 2020;48(4): 420-2. https://doi.org/10.1016/j.ajic.2019.08.006 PMID: 31519479.*

*16.Ofstead CL, Wetzler HP, Johnson EA, Heymann OL, Maust TJ, Shaw MJ. Simethicone residue remains inside gastrointestinal endoscopes despite reprocessing. Am J Infect Control. 2016;44(11):1237-40.https://doi.org/10.1016/j.ajic.2016.05.016PMID: 27497824.*

*17.Ofstead CL, Heymann OL, Quick MR, Eiland JE, Wetzler HP. Residual moisture and waterborne pathogens inside flexible endoscopes: Evidence from a multisite study of endoscope drying effectiveness. Am J Infect Control.2018;46(6):689-96.https://doi.org/10.1016/j.ajic.2018.03.002PMID: 29609854.*

*18.Wallace, M.M,Keck, T,Dixon, H,Yassin, Ml.Borescope examination and microbial culture results of endoscopes in a tertiary care hospital led to changes in storage protocols to improve patient safety.Am J of Infect Control.2023;51(4):361-6. https://doi.org/10.1016/j.ajic.2022.09.009PMID:36122631*

***Reviewer #4: Add duration of the study, location of the study, institutional approval in the part of Settings.***

Response:

Thanks for your suggestion. We have rewritten this part according to the Reviewer's suggestion. The details of the study have been added to make the settings more detailed:*”This study was conducted from May to August 2023 at our CSSD. The project was approved by the medical ethics committee of Henan Provincial People's Hospital (Ethical Clearance No. 125 of 2023).”(P4, Line 73-75)*

***Reviewer #5: It should be passive voice.***

Response:

We apologize for the language problems in the original manuscript. We have rewritten this part according to the suggestion. *(P5, Line 78-79)*

***Reviewer #6: Why not enzymatic detergent?***

Response:

We feel great, thanks to your professional review work on our article. The use of enzyme-containing detergents in intraocular equipment has long been a controversial practice. Firstly, enzymatic detergent typically removes huge amounts of bioburden from surgical instruments, which are smaller in intraocular surgical instruments during surgery than conventional surgical instruments. Secondly, The position of Guidelines for the Cleaning and Sterilization of Intraocular Surgical Instruments advises that minimal bioburden can be completely removed with prompt manual rinsing and cleaning. It is unnecessary to use enzyme detergents routinely. Lastly, given the risk of TASS caused by inadequate rinsing with enzymatic detergent, our hospital's CSSD uses a neutral cleaning agent. This agent is a special design for ophthalmic instruments without enzymes. In response to your reminder, we updated "enzyme-free detergent" to " *a neutral pH detergent (YOMA)*" in the original article. *(P5, Line 89)*

***Reviewer #7: Need guideline or references of this cleaning procedure.***

Response:

Thank you for your reminder. We have checked the literature carefully and added references on this cleaning procedure. *(P5, Line 85)*

***References***

*21.Zhang Q, Huang Hao. Ophthalmic surgical instruments cleaning disinfectant sterilization technology operation guide.Bei Jing:Beijing Science and Technology Press,2016,7:46-8.*

***Reviewer #8: All should be in past tense.***

Response:

We apologize for the language problems in the original manuscript. We have rewritten this part according to the suggestion. *(P5, Line 79-96)*

***Reviewer #9: Define borescope first in the part of Borescope Inspection.***

Response:

Thanks for your suggestion. We have rewritten this part according to the Reviewer's suggestion. The definition of the borescope have been added to make this part more detailed:” *A borescope is a visual inspection system that utilizes a thin, flexible wire equipped with a camera and light source to detect residual materials and contamination within luminal instruments [22.*]”*(P5, Line 99-101)*

***References***

22. *Zhou H.,Shi QF,Gao XD,Wang ZC,Liu XH. Evaluation of cleaning effect of loaner luminal surgical instruments by endoscopic lumen visual inspection system.Chin J Infect Control.2022;21(07):656-60.https://doi.org/10.12138/j.issn.1671-9638.20222668*

**Reviewer #10: Where is the images of inspection procedure.**

Response:

Thanks for your suggestion. We have supplemented the images of the inspection program in the revised manuscript. *The Fig 1 has been added in the Line 116 of P6.*

***Reviewer #11: Should be past tense.***

Response:

We apologize for the language problems in the original manuscript. We have rewritten this part according to the suggestion. *(P5-6, Line 101-112)*

***Reviewer #12: Add reference.***

Response:

Thank you very much for the suggestion. The cleaning procedure for the project's phaco handpiece is based on the instrument manufacturer's instructions as well as the Ophthalmic Surgical Instrument Cleaning and Disinfectant Sterilization Technical Practice Guidelines, which have been included in the references section. Thank you very much for the reminder. *(P5, Line 85)*

***Reviewer #13: Add image of the device.***

Response:

Thanks for your suggestion. We have supplemented the image of the device in the revised manuscript. *The Fig 2 A) has been added in the Line 138 of P7.*

***Reviewer #14: Mention model, origin, quality certification.***

Response:

Thanks for your suggestion. To be more clear and in accordance with the reviewer's concerns, we have added a detailed description as follows:*" The 3M Clean Trace NGi ATP biofluorescence detector was utilized in this study, which exhibited high repeatability and sensitivity, and had been validated by an independent third-party body in the UK. The standard operating procedure employing a 3M™ Clean-Trace™ liquid sampling swab was followed. “(P6, Line 124-128)*

***Reviewer #15: Please add image of working procedure.***

Response:

Thanks for your suggestion. We have supplemented the images of the working procedure in the revised manuscript. *The Fig 2 has been added in the Line 138 of P7.*

***Reviewer #16: Add reference.***

Response:

Thank you for your reminder. We have checked the literature carefully and added references on this testing protocol as follows:" *According to the testing protocol, a reading of ≤150 Relative Light Units (RLU) indicated satisfactory cleaning [24].”(P7, Line 136)*

***References***

*24.3M Health Care. Clean trace ATP surface test: surgical instrument implementation guide for routine cleaning monitoring. Available from: https://www.3m.com/3M/en_US/company-us/all-3mproducts/»/3M-Clean-Trace-ATP-Surface-Test/?N=5002385+3294796486&rt=rud.*

***Reviewer #17: Where is sample size calculation, statistical analysis information?***

Response:

Thanks for your suggestion. We have rewritten this part according to the Reviewer's suggestion as follows: *"Statistical analyses were performed using IBM SPSS Statistics software (version 26.0). Normally distributed continuous variables are reported as Mean ± SD and compared across multiple groups using ANOVA. Categorical variables are reported as frequencies with percentages, with between-group differences assessed using Chi-square tests. A significance threshold of P<0.05 was set to determine statistical significance.” (P7, Line 142-147)*

***Reviewer #18: Simultaneously graphical representation should be demonstrated (Table 1).***

Response:

Thank you for the reminder. We have supplemented the images in the manuscript.

***Reviewer #19: Add graphical representation (Table 2).***

Response:

Thank you for the reminder. We have supplemented the images in the manuscript.

***Reviewer #20: Image should be added for the satisfactory result also.***

Response:

Thank you so much for the suggestion. While tables are more intuitive than text, our team think that including images would distract from readability, given the result of cleaning of each lumen of the phaco handpiece used in this project are good. We truly hope you will accept the expression used in the original article. Thanks!

***Reviewer #21:What’s wrong with enzymatic detergent?***

Response:

We feel great thanks to your professional review work on our article. There is nothing wrong with enzymatic detergent as long as CSSD staff can ensure a thorough rinse. Using enzyme-containing detergents in intraocular devices has been a controversial practice that has not yet reached a uniform view. Therefore, the sterilization supply center can choose the most suitable cleaning agent for ophthalmic surgical instruments according to the conditions of its medical institution, as long as it follows the manufacturer's instruction manual and professional guidelines for ophthalmic surgical instruments. At our hospital, we followed the phaco handpiece manufacturer's instructions and chose a neutral cleaning agent. The exact reason for this has been detailed in question 6. To minimize misinterpretation by readers, we have changed the term “the avoidance of enzyme-based detergents” to *“using suitable detergents* ” to make the expression more accurate.(*P9, Line 199-200*)

***Reviewer #22: For removal of biofilm, enzymatic cleaner is needed.***

Response:

Thank you so much for your question. Biofilm formation has long been challenging for instrument cleaning. The study points out that multiple inappropriate reprocessing cycles could lead to a buildup of residue and microorganisms on instrument surfaces, promoting biofilm formation[1,2]. Enzymatic cleaners can help remove biofilm, but it is also only one of the factors that affect the quality of the cleaning. Currently, there are cleaning solutions specifically developed to remove biofilm, but in practice, or when considering the instrument's structural characteristics itself, choose the suitable cleaning agent. Using enzyme-containing detergents in intraocular devices has been a controversial practice that has not yet reached a uniform view. The original article intended to explain that the rust on the surface of the instruments offers favorable circumstances for biofilm growth and necessitates special attention. The research team has not yet collected reports of biofilm formation inside the lumen of the phaco handpiece, but this is something we will look at in the future. Thanks again for your professional guidance.

**References**

[1]Alfa MJ, Nemes R. Inadequacy of manual cleaning for reprocessing single-use, triplelumen sphinctertomes: Simulated-use testing comparing manual with automated cleaning methods. Am J Infect Control 2003;31(4):193-207.

[2]Vickery K, Pajkos A, Cossart Y. Removal of biofilm from endoscopes: evaluation of detergent efficiency. Am J Infect Control 2004;32(3):170-6. DOI:

10.1016/j.ajic.2003.10.009

***Reviewer #23: Should be passive.***

Response:

We apologize for the language problems in the original manuscript. We have rewritten this part according to the suggestion. *(P10, Line 224-226)*

***Reviewer #24: Passive, add reference.***

Response:

We apologize for the language problems in the original manuscript. We have rewritten this part according to the suggestion. There is no reference for this sentence. To avoid confusion for readers, we changed the original text as "*As shown in Table 1, initial lumen-related damage was observed at a mean service duration of 6.06±1.03 years (Mean ± SD). Based on these findings, we recommend routine inspections for handpieces exceeding five years of clinical use, with a particular focus on irrigation lumen integrity. This proactive maintenance approach allows for the early detection of structural deterioration, ensuring patient operational safety for patients.’’ (P10, Line 233-238)*

***Reviewer #25: Why the ATP results were satisfactory? What precautions were taken?***

Response:

Thank you very much for your professional advice. To be more clear and in accordance with the reviewer's concerns, we have added a detailed description as follows:" *ATP bioluminescence testing yielded satisfactory results, confirming the efficacy of our standardized decontamination protocols, which align with the manufacturer specifications and evidence-based guidelines from ophthalmic professional associations. However, as ATP bioluminescence detects residual organic contamination detection, its sensitivity is limited when applied to phaco handpieces where bioburden levels remain low. Furthermore, ATP bioluminescence is less effective than borescope inspection for detecting structural damage. Routine borescope examinations revealed persistent structural defects that, despite meeting cleanliness standards, could serve as potential biofilm reservoirs [25]. These findings underscore the critical need for a multimodal quality assurance strategy. We propose implementing a comprehensive surveillance system that monitors microbial contamination and device integrity.” (P11, Line 239-249)*

***Reviewer #26: No cleaner information is given about phaco hand piece sterilization practice.***

Response:

Thank you very much for your professional advice, and we will give more about phaco handpiece clean practice in the article as follows:" *This study demonstrates previously unrecognized cleaning challenges and highlights three key preventive measures: 1) prompt post-surgery irrigation of instrument channels with sterile distilled water, 2) implementation of controlled-environment protocols for compressed-air drying to minimize particulate contamination, and 3) establishment of scheduled maintenance cycles to preserve lumen integrity. (P11, Line 252-256)*

***Reviewer #27: The conclusion is too short.***

Response:

Thank you very much for your professional advice, which we will add further in our conclusions as follows:*”This study highlights the value of borescope inspection as part of a visual inspection of phaco handpiece integrity, identifying issues such as corrosion and rust that necessitated immediate lumen flushing with sterile distilled or deionized water during surgery. The detection of green lint prompted the discontinuation of textile fiber-containing wipes on the operating table, thereby enhancing patient safety measures. Future advances are expected to integrate both qualitative and quantitative approaches for a more comprehensive evaluation of instrument cleanliness. Additionally, ongoing efforts will focus on mitigating corrosion and particulate formation during rinsing to further enhance the effectiveness of cleaning protocols.” (P11-12, Line 264-272)*

Finally, in addition to the above revisions, the research team rechecked the full text and made the following revisions: 1) the graphs and tables of the article were rearranged and passed the PACE system; 2) the references were reorganized according to the revisions; 3) the data were uploaded to the public databases(https://doi.org/10.6084/m9.figshare.28466747.v1); and 4) the ethical review documents were supplemented. We thank the experts again for their professional guidance on the article. Thanks very much for your attention to our paper.
